# MiR-326 inhibits trophoblast growth, migration, and invasion by targeting PAX8 via Hippo pathway

**DOI:** 10.1186/s12958-022-00909-2

**Published:** 2022-02-24

**Authors:** Junjie Zang, Min Yan, Yan Zhang, Wei Peng, Jianxin Zuo, Huansheng Zhou, Guoqiang Gao, Min Li, Yijing Chu, Yuanhua Ye

**Affiliations:** 1 Department of Obstetrics, the Affiliated Hospital of Qingdao University, 16 Jiangsu Road, 266000 Qingdao, China; 2 Department of Obstetrics, the Weifang Yidu Central Hospital, Weifang, China

**Keywords:** miR-326, PAX8, Trophoblasts, Hippo signaling

## Abstract

Preeclampsia (PE), a pregnancy disorder that affects 5–7% of pregnant women, is among the primary causes for maternal and perinatal mortality. PE is believed to be associated with insufficient invasion of villous and extravillous trophoblasts (EVTs), which hampers uterine spiral artery remodeling and finally induces PE. But the mechanism responsible for reduction of trophoblast invasion remains unclear. In this study, placental tissues taken from healthy donors and PE patients were used to evaluate the miR-326 expression; CCK8 and colony formation assays were used to confirm the effect of miR-326 on cell proliferation; transwell assay was used to demonstrate the effect of miR-326 on cell invasion capability; western blot was used to investigate the underlying mechanism; and luciferase assay was used to detect the effect of miR-326 on YAP/TAZ-mediated transcription activity. It was revealed the miR-326 expression was higher in placentas from PE patients than from healthy donors. After transfection of miR-326 mimics, trophoblast proliferation and invasion were impaired. Using TargetScan, we speculated that PAX8 was a target of miR-326, which was later confirmed by western blot. The YAP/TAZ expression was also downregulated after transfection with miR-326. Luciferase assay demonstrated that overexpression of miR-326 suppressed YAP/TAZ-mediated transcription activity by targeting PAX8. Overexpression of PAX8 could partly rescue miR-326-induced suppression of trophoblast proliferation and invasion. Taken together, our result indicated that miR-326 suppresses trophoblast growth, invasion, and migration by means of targeting PAX8 via the Hippo pathway.

## Introduction

PE is typically symptomized by high levels of blood pressure and urine protein, and accounts for about 5% of pregnancies [1]. PE occurs usually 20 weeks into gestation, and is determined as the major cause for premature delivery, placental abruption, low infant weight, and perinatal and maternal fatality [2]. Although the exact pathophysiology of PE is largely unknown, aberrant placental development is widely recognized as initiatory symptom during early gestation [3]. Trophoblasts, which are major cellular components of placenta, play essential roles in the functioning of placenta. Trophoblast viability and invasion have been reported to be closely correlated with the PE progression [4].

MicroRNAs (miRNAs) are short, noncoding RNA sequences involved in the regulation of gene expression by suppressing translation or activating degradation of its target mRNA [5]. miRNA dysregulation is observed in various diseases, including malignant cancers and immunological disorders [6–9]. Recent evidences have showed that miRNAs act as critical molecules in the PE development [10, 11]. miRNAs were differentially expressed in trophoblasts of both mild and severe PE patients. They specifically targeted genes that are involved in signal transductions, resulting in alteration of processes that are directly related to the PE progression [12, 13]. Trophoblast proliferation and invasion, angiogenesis and other distinct characteristics of placentation, are regulated to different extents by differential miRNA expressions. miR-326 has been reported to suppress the progression of cancer by regulating proliferation signal transduction in cancer cells [14]. The miR-326 expression in placentas both from normal and PE subjects were examined, and the mechanism of how miR-326 affected PE progression was investigated in this study.

## Methods and materials

### Patients and samples

All experiments and use of placentas were approved by the Ethical Committee of the Affiliated Hospital of Qingdao University (Shandong, China). All participants had given informed consents before any experiment. Placental samples were either acquired from normal full‑term delivery (n = 6, weeks 37–39) or from severe PE patients (n = 6, weeks 37–39), both by parental permissions. Exclusion criteria were multiple pregnancies, pregnancies with fetal anomalies, pregnancies with chronic disease, preexisting hypertensive disorders, chorioamnionitis, diabetes mellitus, and gestational diabetes. All placentas were harvested by elective cesarean section without involving artificial labor. Placental tissues were extracted by cutting a vertical plane across a central and normal area, including both the fetal and maternal sides. Tissues involving calcification or clots were discarded.

### Cell Culture

To fully understand the role of miR-126 in trophoblast functions, we used two trophoblast cell lines (HTR-8/SVneo and JEG-3, Type Culture Collection China Centre) in this study. Both cell lines were cultured with DMEM/F12 (including 10% FBS) in an incubator at 37 °C in an atmosphere with 5% CO_2_.

### Small interfering RNA, plasmids, miR mimic/inhibitor, and lentivirus transfection

Cell transfection was conducted on Lipofectamine 2000 (Invitrogen, Carlsbad, USA). Then these transfected cells were isolated as single clones following puromycin treatment to construct stably transfected cell lines.

The adopted shRNA sequences are shown as below: sh-NC, 5’- GCAAGCTGACCCTGAAGTT − 3’; sh-PAX8, 5’- GCCAGAACCCTACCATGTT-3’;

Control miRNA, and miR-326 mimics and inhibitors were acquired from Hanbio (Shanghai, China).

Lentivirus control or lentiviral constructs with full PAX8 expression and miR-326 overexpression were adopted to construct stably transfected cell lines.

### Western blot analysis and quantitative real-time PCR (qRT-PCR)

Western blot was conducted as previously desgribed [15]. The following primary antibodies were adopted in the analysis: PAX8 (ab53490, Abcam, Cambridge, MA; 1:1000), TAZ (ab205270, Abcam; 1:1000), YAP (ab52771, Abcam; 1:1000), GAPDH (ab181603, Abcam; 1:5000).

The primers were purchased from Shanghai Genechem Co.,Ltd, and the qRT-PCR assays were performed as previously described [16]. The primer sequences were as follows: PAX8, forward, 5’-GATCCTCACTCACCCTTCGC-3’, reverse, 5’-TAACCACACAGGGAGTGTGC-3’; GAPDH forward, 5’- AATGGGCAGCCGTTAGGAAA-3’, reverse, 5’- GCGCCCAATACGACCAAATC-3’; miR-326, forward, 5’- ACTGTCCTTCCCTCTGGGC − 3’, reverse, 5’- AATGGTTGTTCTCCACTCTCTCTC − 3’; U6 forward, 5’- CTCGCTTCGGCAGCACA − 3’, reverse, 5’-TGGTGTCGTGGAGTCG − 3’.

### Cell Proliferation Analysis, transwell assays and colony formation assays

Cell proliferation analysis, transwell assays and colony formation assays were carried out as previously specified [15]. We added trophoblasts to ninety-six-well plates (density: 5,000 cells per well), cultured these cells, and measured trophoblast proliferation daily via CCK-8 assays (Thermo Fisher Scientific, MA, USA). We added CCK-8 reagent to each well, and the trophoblasts were cultured for an additional 1.5 h. Then, colourimetric assays were performed by measuring the OD value of each well in a microplate reader (wavelength: 450 nm). Growth curves were determined in three independent experiments.

For transwell invasion assay, serum-free cell suspension culture (4 × 10^4^) were plated on the upper chamber membranes coated with Matrigel (BD Biosciences). Then, 600 µl medium (including 10% FBS) was supplement into the lower chamber. After 24 h, the lower side of membrane was fixed for 30 min and then subjected to crystal violet (0.1%) staining. The inner side of membrane was swabbed using a cotton swab, and cells quantification was performed with a microscope.

For colony forming assay, 500–1000 trophoblasts were incubated in 6-well plates for 14 days. Paraformaldehyde was then used to fix the colonies which were afterwards stained with a crystal violet solution for visualization.

### Luciferase reporter assays

Cells (3 × 10^3^ for each well) were plated in 96-well plates. Plasmids with pGL3- PAX8 3’ UTR WT (wild-type) or pGL3- PAX8 3’ UTR MUT (mutant) were synthesized by Hanbio (Shanghai, China). Co-transfection of the plasmids and miR-326 mimics or inhibitors was performed using Lipofectamine3000 (Thermo Fisher Scientific).

Cells (5 × 10^3^ for each well) were plated in 96-well plates. Co-transfection of synthetic TEAD luciferase reporter (Addgene, Cambridge, USA) and Renilla reporter constructs (Promega; Madison, USA) into stably transfected cells was performed on Lipofectamine 3000. Normalization of Luciferase reporter activity was performed with Renilla luciferase. After 24 h, luciferase activity was measured by a Dual-Luciferase Reporter Assay Kit (Promega).

### Statistical analysis

All data were processed by unpaired two-tailed t-test. Each analysis was carried out at least in triplicate and all data are reported as the mean ± SEM. GraphPad Prism (GraphPad; La Jolla, USA) was used in data analysis. *P* value < 0.05 (expressed as “*”) suggested statistical significance; *P* value < 0.01 were denoted as “**” and *P* value < 0.001 was denoted “***”. *P* value > 0.05 (expressed as “n.s.”) was defined as statistically insignificant.

## Results

### miR-326 inhibits trophoblast proliferation and invasion

**Fig. 1:**
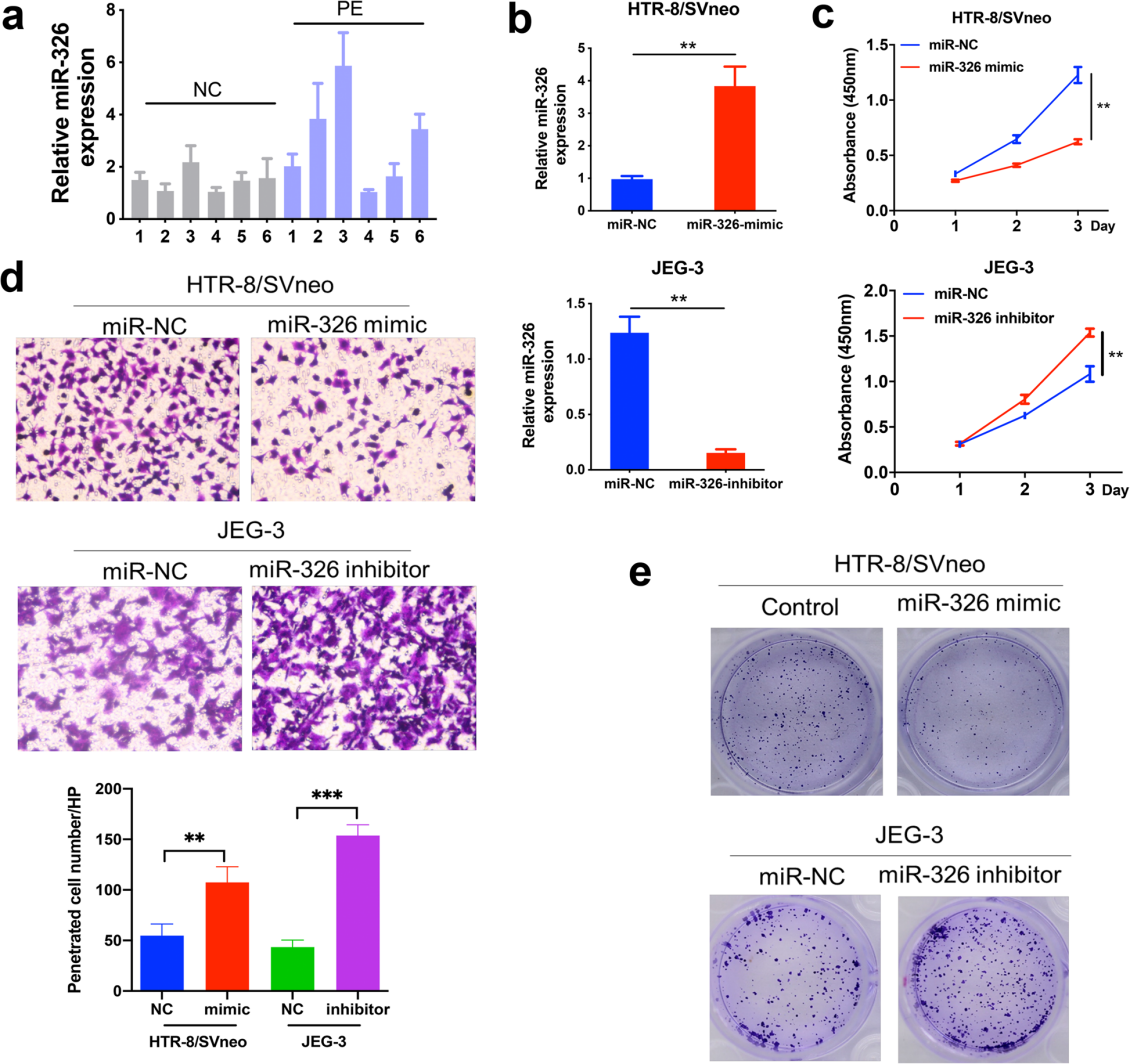
miR-326 inhibits cell proliferation, migration and invasion. **a** MiR-326 levels in placenta tissues from pregnant women with PE or normal controls was detected by qRT-PCR. **b** MiR-326 levels in trophoblasts transfected with MiR-326 mimics or inhibitor was detected by qRT-PCR. GAPDH was used for normalization. **c** CCK8 assays from indicated groups to evaluate cell proliferation. **d** Transwell assays to evaluate cell migration and invasion. **e** Colony formation assays in cells treated with miR-326 mimics or miR-326 inhibitors

First, we detected miR-326 levels in placentas from both healthy donors and PE patients by qRT-PCR, and found that the relative expression of miR-326 was significantly higher in placentas from the PE patients (Fig. 1a). Then, transfection of miR-326 mimics/inhibitors as well as corresponding negative controls into trophoblasts was performed and lasted for 48 h. It was noticed that miR-326 expression in trophoblasts was substantially upregulated by miR-326 mimics and downregulated by its inhibitors (Fig. 1b). In addition, it was observed that trophoblast proliferation and invasion as well as its colony formation capability were inhibited by overexpression of miR-326 and advanced by inhibition of miR-326, respectively (Fig. 1c-e). These findings suggested that upregulation of miR-326 in placentas of PE patients could inhibit trophoblast proliferation and invasion.

### PAX8 is a potential target of miR-326 and a promoter of trophoblast proliferation and invasion

**Fig. 2:**
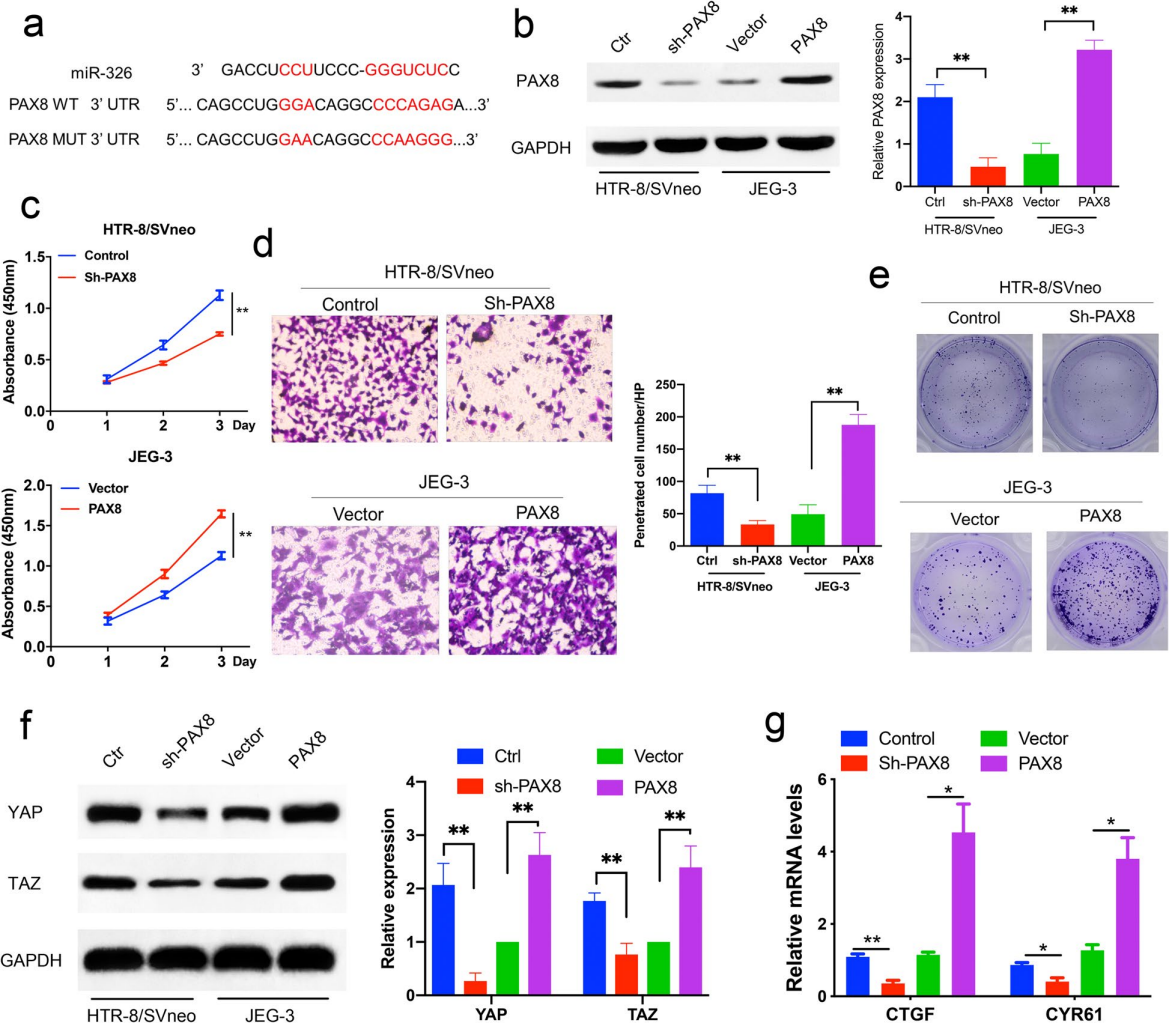
PAX8 promotes proliferation, migration and invasion of trophoblast cells. **a** Schematic diagram of miR-326 and its putative binding sequence in the wild-type (WT) and mutant (MUT) 3′-UTR of PAX8. **b** Western blot analysis to validate the efficiency of stably transfected cells. **c**, **d**, **e** CCK8 assays, transwell assays and colony formation assays from indicated cells with PAX8 knockdown or overexpression **f** Western blot analysis for YAP and TAZ protein in indicated cells. **g** Relative Hippo pathway activity. Renilla activity was used to normalize luciferase reporter activity

miRNAs have long been recognized as target-gene regulators in cells. We found PAX8 was a potential target of miR-326 by bioinformatic analysis of data from microRNA.org (http://www.microrna.org/microrna/), miRDB (http://mirdb.org/cgi-bin/), and TargetScan (http://www.targetscan.org/) database. The results suggested that 3’-UTR of PAX8 bound to the binding site of miR-326 (Fig. 2a). To further determine if the PAX8 expression could affect trophoblast proliferation and invasion, trophoblasts were transfected with the PAX8 siRNA or PAX8 overexpression plasmids. The western blot analysis confirmed the inhibitory/promoting effects of PAX8 shRNA/ overexpression lentivirus on the PAX8 levels in trophoblasts (Fig. 2b). In addition, we examined the proliferation of trophoblasts transfected with PAX8 siRNA or PAX8 plasmids by CCK-8 assays. It was shown that PAX8 overexpression raised the trophoblast proliferation rate, and proliferation suppression was observed in trophoblasts transfected with PAX8 siRNA (Fig. 2c). In consistence with the findings in CCK-8 assays, transwell assays and colony formation assays also revealed that PAX8 overexpression significantly enhanced trophoblast invasion and their colony formation, while PAX8 downregulation induced opposite effects (Fig. 2d-e). Our results suggested that PAX8, which is directly targeted by miR-326, could regulate the proliferation, invasion and colony formation of trophoblasts.

It has been reported that PAX8 serves as a nuclear transcription factor for a variety of genes. As the main effector molecule downstream of Hippo pathway and two related transcription regulators in biological regulation function, it has been revealed that YAP/TAZ had a low expression in PE [17]. We thus speculated that PAX8 might be involved in regulation of YAP/TAZ activity. As shown in Fig. 2f, the expression of YAP/TAZ was suppressed following PAX8 downregulation, and increased after PAX8 overexpression. This implied that PAX8 could regulate YAP/TAZ expression in trophoblasts. YAP/TAZ is classical transcriptional factor that promotes gene transcription. Two typical YAP-targeted genes, CTGF and CYR61, were used to investigate the effect of PAX8 on YAP/TAZ functions. As shown in Fig. 2 g, PAX8 downregulation inhibited the mRNA expressions of CTFF and CYR61, and PAX8 overexpression had opposite effects.

Taken together, our result indicated that miR-326 act through PAX8 in trophoblasts; it could promote trophoblast proliferation and invasion, and regulate YAP/TAZ activity.

### PAX8 is a direct miR-326 target in trophoblasts

**Fig. 3:**
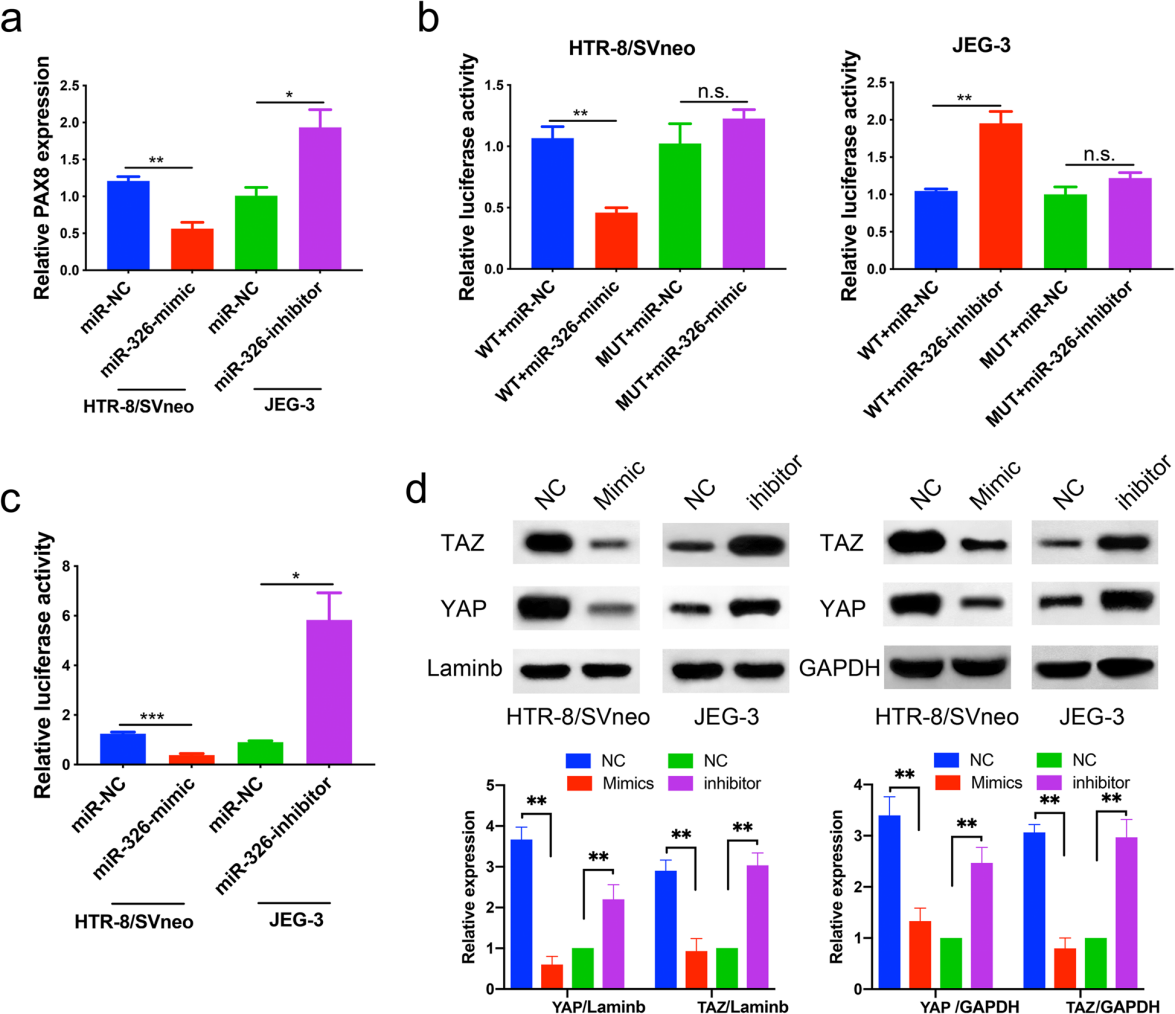
PAX8 is a direct target of miR-326 in trophoblasts. **a** qRT-PCR analysis of PAX8 mRNA expression in cells treated with miR-326 mimic or miR-326 inhibitor. **b** Relative luciferase activity in indicated cell groups. **c** Relative Hippo pathway activity with different treatments. **d** The levels of YAP and TAZ in nucleus or cytoplasm of trophoblasts in indicated groups were tested by western blotting

To further confirm that PAX8 was directly targeted by miR-326, we first evaluated the mRNA levels of PAX8 in trophoblasts by qRT-PCR after transfection with miR-326 mimics or inhibitors. miR-326 overexpression in trophoblasts substantially upregulated the mRNA level of PAX8, whereas miR-326 inhibition upregulated it, as shown in Fig. 3a. Luciferase assays were then conducted. The results of assays in HTR-8 cells showed that miR-326 mimics significantly reduced the relative activity of luciferase with wild-type 3’-UTR of PAX8 (*P* < 0.01), while treatment of miR-326 inhibitors in JEG-3 cells did not noticeably affect the activity of luciferase with mutant 3’-UTR of PAX8 (Fig. 3b). In addition, miR-326 inhibitors considerably increased the luciferase activity with wild-type 3’-UTR of PAX8 in JEG-3 cells (*P* < 0.01, Fig. 3b).

The Hippo pathway is an evolutionarily-conserved pathway that coordinates cell proliferation and apoptosis to regulate organ sizes [18]. We examined the relative luciferase activity in the Hippo pathway in trophoblasts transfected with miR-326 mimics and inhibitors. The sequence of YAP binding site was constructed upstream of luciferase, and thus the YAP/TAZ activity can be directly monitored by the luciferase activity. It was revealed that miR-326 mimics substantially reduced the relative luciferase activity in the Hippo pathway in HTR8 cells (*P* < 0.01), while miR-326 inhibitor treatment in JEG-3 cells promoted relative luciferase activity (*P* < 0.05, Fig. 3c). Then, we examined the YAP/TAZ expression in trophoblasts treated with miR-326 mimics or inhibitors by western blot. The results showed that the miR-326 overexpression could significantly inhibit the YAP/TAZ expression in trophoblast nucleus, and miR-326 inhibition could promote the nuclear expression of YAP/TAZ (Fig. 3d).

Taken together, we revealed that miR-326 can regulate the Hippo pathway activity by targeting PAX8.

### MiR-326 reduces cell proliferation, migration and invasion by targeting PAX8

**Fig. 4:**
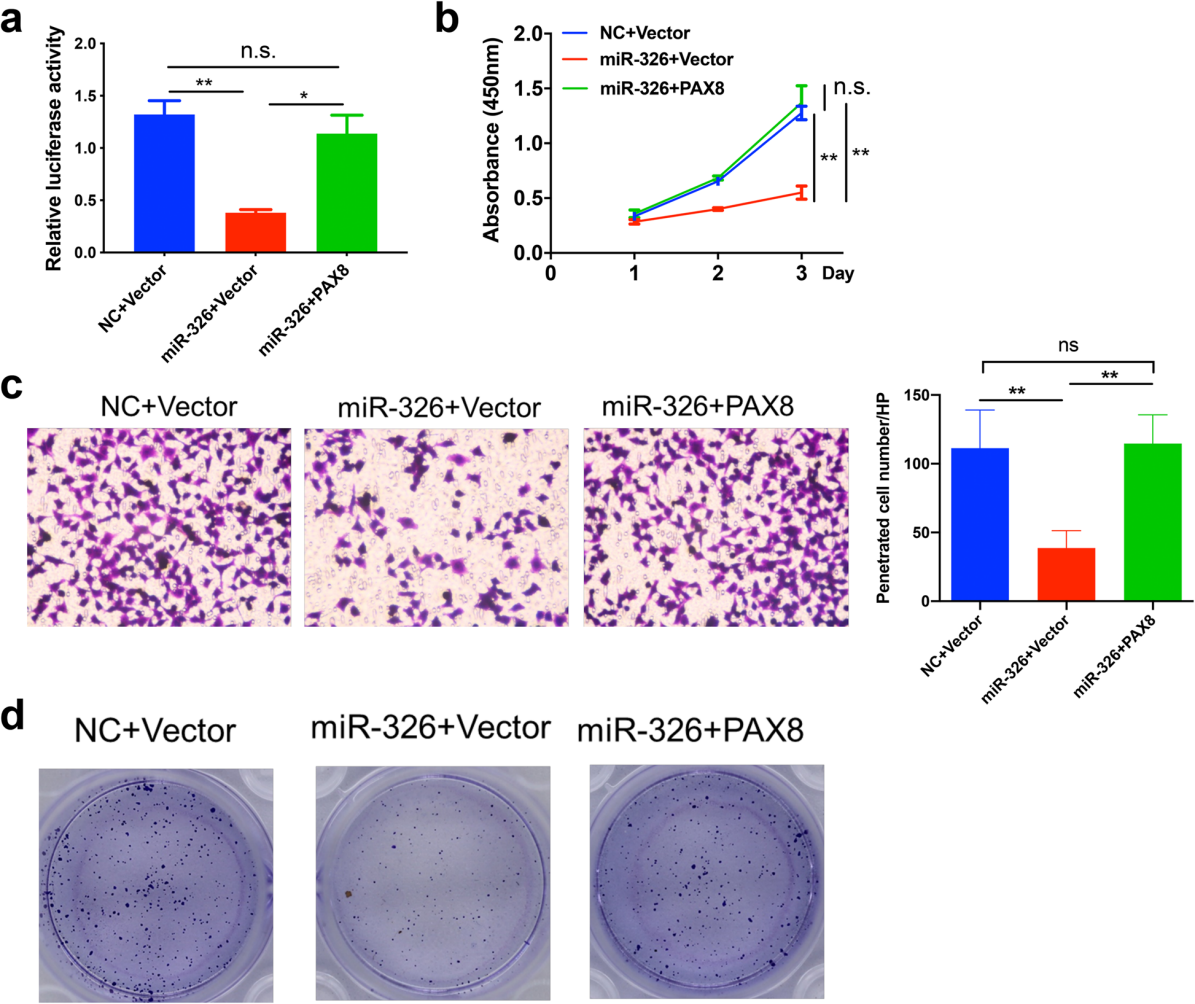
miR-326 reduces cell proliferation, migration and invasion by targeting PAX8. **a** Relative Hippo pathway activity to validate the efficiency of stably transfected cells. **b**, **c**, **d** CCK8 assays, transwell assays and colony formation assays from indicated cells

Given the previous finding that miR-326 can regulate the Hippo pathway activity, we further investigated whether such effect was dependent on the PAX8 regulation. As shown in Fig. 4a, after transfection of miR-326, the Hippo pathway activity was significantly suppressed as expected, and after overexpression of PAX8, the Hippo pathway activity was partly rescued, indicating that suppression of the Hippo pathway activity induced by miR-326 is dependent on the downregulation of PAX8.

Next, we examined if the effect of miR-326 on cell proliferation or invasion relied on the regulation of PAX8 expression. As shown in Fig. 4b-d, the proliferation and invasion of trophoblasts were suppressed after transfection of miR-326 as previously mentioned. However, after transfection of the PAX8-overexpression plasmid, the proliferation and invasion capability was partly rescued, suggesting that miR-326 suppressed trophoblast proliferation and invasion through the PAX8 regulation.

Taken together, our result indicated that miR-326 reduces cell proliferation, migration, and invasion by targeting PAX8.

## Discussion

PE is a major complication of pregnancy symptomized by hypertension and proteinuria [19–23]. At the first trimester of pregnancy, the invasive EVTs induce the extension of maternal spiral arteries. Impaired EVT invasion, accompanied with poor spiral vascular remodeling, induces inadequate placental perfusion, and subsequently results in pathologic hypoxia and release of soluble factors, leading to maternal blood pressure elevation. Therefore, the pathogenesis of PE is associated with the decrease in trophoblast invasion, which is also observed in tumors [24–27]. miRNAs are single-stranded, noncoding RNA sequences containing approximately 22 nucleotides, which are evolutionarily conserved, and exert their regulations by binding to the 3’UTR of target genes and inhibiting their expression [28–30]. Moreover, several studies have reported that miRNAs, such as miR-326, play vital roles in the cellular processes of cancers. A variety of genes could be involved in regulations of cell apoptosis and proliferation through miR-326, including fibroblast growth factor 1 (FGF1), CyclinD1 (CCND1) and neuroblastoma RAS viral oncogene homolog (NRAS) [31]. Although a few studies have revealed that miRNAs are crucial regulatory elements during pregnancy, their exact roles in PE development remain largely unknown. In this study, we demonstrate that miR-326 may play a role in the pathogenesis and progression of PE. It is firstly observed that placentas from PE patients have a higher expression of miR-326 than those from healthy donors. Secondly, inhibition of trophoblast proliferation and invasion was observed after miR-326 overexpression, indicating that miR-326 overexpression in trophoblasts may suppress their invasion and thus induce the development of PE. This finding could provide an innovative means for PE diagnosis and management.

PAX8 a member of paired-box family that is expressed during organogenesis of kidney, thyroid and Mullerian tract [32, 33]. PAX8 transcription factor plays a critical role in viability of differentiated epithelial cells, and overexpression of PAX8 is associated with development of different types of tumors [34]. TAZ and YAP, the downstream effectors of Hippo signaling, can stimulate cell growth, migration, invasion and immune escape, which leads to in-vivo tumor development and therapy resistance [35]. The Hippo pathway is involved in many pathophysiological processes, including cell proliferation, migration and invasion, which are closely related to many diseases [36]. Interestingly, the Hippo signaling is also implicated in the pathological process of PE [17]. It was confirmed in this study that trophoblast invasion and proliferation were significantly suppressed after PAX8 knockdown. It was also revealed that PAX8 downregulation suppressed the expression of YAP/TAZ. Our data also showed that miR-326 may regulate the PAX8 expression to affect the hippo signaling pathway.

Nevertheless, this study has some limitations. First, investigations were only conducted in placental tissues and trophoblast cell lines, and animal models should be required in further researches. Efforts should be made in comparing miR-326 expressions in pregnancies of mice in the future. Second, primary trophoblast cells were not investigated in placentas from those who would or would not have manifestations of PE in the early pregnancy. Third, in comparison with the same pregnancy week of control group, the amount of PE blood sample was small, and further studies with specimen enlargements should be performed. In addition, the miR-326 level in one PE patient was very low, and there may be interference from other factors.

To summarize, our results indicated that miR-326 suppresses trophoblast proliferation and invasion by targeting PAX8.

## Data Availability

The datasets used and/or analyzed during the current study are available from the corresponding author on reasonable request.
